# *Companion*: a web server for annotation and analysis of parasite genomes

**DOI:** 10.1093/nar/gkw292

**Published:** 2016-04-21

**Authors:** Sascha Steinbiss, Fatima Silva-Franco, Brian Brunk, Bernardo Foth, Christiane Hertz-Fowler, Matthew Berriman, Thomas D. Otto

**Affiliations:** 1 Parasite Genomics, Wellcome Trust Sanger Institute, Hinxton, Cambridge CB10 1SA, UK; 2 Institute of Integrative Biology, University of Liverpool, Liverpool L69 7ZB, UK; 3 Department of Biology, University of Pennsylvania, Philadelphia, PA 19104, USA

## Abstract

Currently available sequencing technologies enable quick and economical sequencing of many new eukaryotic parasite (apicomplexan or kinetoplastid) species or strains. Compared to SNP calling approaches, *de novo* assembly of these genomes enables researchers to additionally determine insertion, deletion and recombination events as well as to detect complex sequence diversity, such as that seen in variable multigene families. However, there currently are no automated eukaryotic annotation pipelines offering the required range of results to facilitate such analyses. A suitable pipeline needs to perform evidence-supported gene finding as well as functional annotation and pseudogene detection up to the generation of output ready to be submitted to a public database. Moreover, no current tool includes quick yet informative comparative analyses and a first pass visualization of both annotation and analysis results. To overcome those needs we have developed the *Companion* web server (http://companion.sanger.ac.uk) providing parasite genome annotation as a service using a reference-based approach. We demonstrate the use and performance of *Companion* by annotating two *Leishmania* and *Plasmodium* genomes as typical parasite cases and evaluate the results compared to manually annotated references.

## INTRODUCTION

The availability, extent and quality of genomic annotations are of crucial importance for powerful genomics methods like comparative studies, expression analysis or even simple gene knockdown (1). To reflect the richness of genomic features that can be annotated, a useful genome annotation should contain a high-quality set of both coding and noncoding gene features, the latter comprising transfer RNA, ribosomal RNA and small nuclear and nucleolar RNA genes as well as pseudogenes. For protein-coding genes, identifying orthologs in a reference species is a key step for comparative analysis aimed at identifying major genic differences between species. Typical characteristics to examine are similarities and differences in gene content, phylogenetic relationships and synteny. To further characterize these differences, functional information about the genes involved is required, encompassing protein product descriptions and controlled vocabulary terms, e.g. for function and localization (2,3). The availability of such results at a researcher's disposal right after the initial annotation helps to determine the direction for subsequent in-depth analyses. Though many comparative efforts have previously focused on resequencing and variant calling, the emergence of long read sequencing technologies makes generating *de novo* assemblies technically easier. The use of annotated full genomes is more powerful to identify new genes and large-scale rearrangements as well as to understand variable regions not covered by mapping approaches. While established ‘out of the box’ software – such as Prokka (4) and RAST (5) – exists for extensively annotating the genomes of prokaryotes, similar software packages for eukaryotes are lacking. For eukaryotic genomes, many tools exist to perform the basic task of *ab initio* gene finding (6–9), optimized to accurately predict the boundaries of all genes and their exons in the genome sequence. Most of these tools use machine-learning approaches that require training with manually curated gene models and/or extrinsic evidence such as RNA-seq transcripts. Another related but challenging task is to correctly call functional genes as opposed to pseudogenes, which show a similar sequence footprint but are not translated. In the next step, a putative function must be ascribed to each new gene. That is generally achieved though similarity searches or transferred through orthology clusters, e.g. using OrthoMCL (10). Another important aspect, and often underestimated yet nontrivial (11), is the generation of a suitable output format for submission to public databases.

To address the demand for quick, automatically generated parasite genome annotations, we have developed *Companion* (COMprehensive Parasite ANnotatION) as a web server. It allows parasitology researchers to upload their target assemblies and select a closely related reference species to guide the annotation. *Companion* delivers a readily usable annotation of features in the target genome, as outlined above, in a variety of different formats including those required for submission to public databases. Moreover, it implements several features to highlight gene content differences between the reference and the new assembly, such as identification of orthologous clusters, species-specific singleton genes and missing core genes present in a larger reference species set. To recognize misassemblies or rearrangements, it also provides a high level visualization of sequence matches. The web server currently offers 62 species to be used as references. The open source pipeline underlying the web server, however, is extensible and can also be run separately, for instance to handle larger input, such as larger parasite genomes, on more powerful systems.

## METHODS

**Figure 1.**
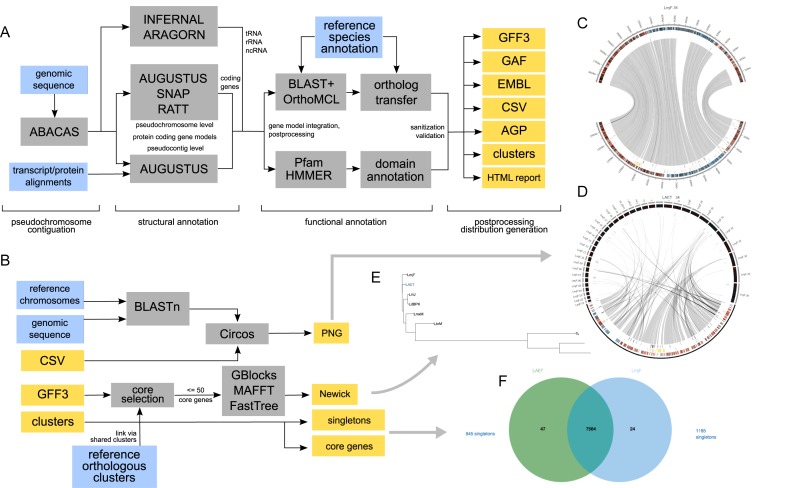
Schematic overview of the *Companion* workflows. (**A**) – genome annotation workflow, (**B**) – downstream analysis and visualization workflow. Input files are represented as blue boxes, output files as yellow boxes. All output files are used to construct the result set presented in the web interface: (**C**) and (**D**) – target-reference synteny diagrams for the *Leishmania aethiopica* target chromosome 34 and the unassembled ‘bin’ chromosome (the latter not drawn to scale), (**E**) – zoomable tree placing the newly annotated species (here ‘LAET’) in the context of the reference species set, (**F**) – interactive Venn diagram summarizing core and species-specific clusters.

This section describes the approach taken to perform the various steps that make up the complete workflow. The workflow can be roughly divided into the phases of contiguation, feature annotation, functional annotation, evaluation and visualization (Figure 1).

### Pseudochromosome contiguation

The first optional step is to order and orientate the input sequences (e.g. contigs or scaffolds) against the reference using ABACAS2 (https://github.com/sanger-pathogens/ABACAS2) to match the chromosome structure of the reference genome as far as possible. Unordered input sequences are concatenated into an additional, artificial ‘bin’ sequence. AGP files describing the resulting chromosome and bin layouts are created for subsequent database submission.

### Annotation workflow

**Figure 2.**
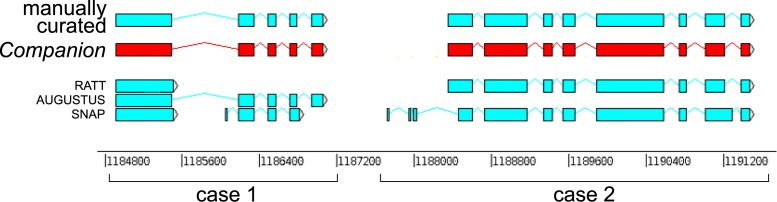
Example of gene model integration across different gene finders. Case 1 depicts a situation in which RATT was not able to correctly produce a sensible gene model. In case 2, AUGUSTUS missed this gene completely.

The structural annotation component uses both homology-based and *ab initio* annotation techniques to deliver a set of protein-coding gene models. RATT (12) is used to transfer highly conserved gene models with little or no modification from the reference to the target. *Ab initio* gene prediction methods such as SNAP (9) and AUGUSTUS (8) are used as an additional source of candidate gene models and make use of extrinsic evidence such as EST or RNA-seq data (13,14) if available. AUGUSTUS models were trained using full coding gene models from the reference data set. In order to reliably identify partial genes flanking gaps, gene finding is performed both on the complete pseudochromosomes as well as the bin. We also perform *de novo* gene prediction on all input sequences split at gaps, allowing AUGUSTUS to call partial genes at the boundaries of each such obtained ‘pseudocontig.’ At the end of the structural annotation step, a final nonredundant set of gene models is obtained by merging the results of all gene finders into a canonical set. This is done by choosing the best explanation for any given locus, depending on source, length and semantic properties such as splice site canonicity (Figure 2).

For each protein-coding gene in the resulting set, functional annotation (product descriptions, gene names, GO terms, IDs of orthologs) is transferred from annotations associated with orthologous reference genes defined by OrthoMCL (10). If no previously characterized orthologs can be determined for a gene, the best Pfam-A hit (15) is used to assign a putative function. All functional data are tagged with GO compliant evidence (e.g. IEA, ISO) and reference (GO_REF) codes.

Pseudogenes are annotated using protein-DNA alignments allowing for frame shifts (16) using the LAST aligner (17). The resulting alignments are combined into superhits using an approach similar to the one employed by PseudoPipe (18). Using a novel reconciliation step, these superhits are then compared to the previously determined gene models using a rule-based approach with the goal of choosing a gene or pseudogene model as the most likely explanation for a given alignment to a locus (see Supplementary file 1).

Additional noncoding RNA annotations are produced *ab initio* by ARAGORN (19) for transfer RNA and INFERNAL (20) with ribosomal RNA and other ncRNA covariance models selected from the Rfam database (21) for other RNAs. Both coding and noncoding gene models are finally merged into one complete structural annotation set.

As the genes of kinetoplastids such as *Trypanosoma* and *Leishmania* are organized into large directional clusters of genes that are transcribed together as polycistrons (22), we implemented a filtering method to eliminate overprediction of genes on the other strand (see supplementary file 1 for details).

### Result preparation and comparative downstream analyses

To facilitate interpretation of the results, the *Companion* pipeline produces an extensive set of result files (see Figure 1A). Sequence and annotation files are available for download in compressed form. Based on the annotations and orthology information produced in the previous steps, *Companion* performs and visualizes various downstream analysis steps to help in the interpretation of the annotation (Figure 1B). *Companion* visualizes the OrthoMCL output, highlighting shared and species specific genes using Venn diagrams (Figure 1F) using JVenn (23). The content of each intersecting subset can be browsed in a paginated and sortable table, enabling interactive analysis of gene content differences. A species tree built from up to 50 randomly sampled orthologous clusters, each with a single copy in each of the organisms, is created using MAFFT (24), Gblocks (25) and FastTree (26). If pseudochromosome contiguation is enabled, per-chromosome synteny plots (27) based on nucleotide matches are produced as well. The resulting plots deliver a concise overview of the reference-target alignment including genes, polycistronic transcription units, assembly features (e.g. gaps), singleton genes and missing core genes. The plots highlight the assembly quality and help to identify errors such as repeat compression or misassembly, as well as identify large-scale chromosomal rearrangement.

### Availability

The *Companion* web interface is available for public use without login requirement at http://companion.sanger.ac.uk. Users are free to submit their own sequences in FASTA, GenBank or EMBL format up to a size of 64 MB and a maximum of 3000 sequences. As a reference set, we provide a diverse set of 62 selected parasite species imported from the latest versions of the GeneDB (28) and EuPathDB (29) databases. For use on confidential or large genome sequences, the *Companion* pipeline is also available on selected platforms, including Linux and Mac OS X, as a stand-alone tool and can be obtained from http://github.com/sanger-pathogens/companion. See Supplementary file 1 for more details.

## EVALUATION RESULTS

To assess the performance of *Companion* in terms of annotation quality and completeness, we have used the stand-alone version of the software to compare the performance on two manually curated genomes. The comparison was performed using ParsEval (30) as well as a custom in-house comparison tool modeled after Eval (31). We first evaluated the impact of different parameter sets on a single chromosome and then compared whole genome annotations (see Supplementary file 1 for more details on the exact measures used). For each of the evaluation species, the curated genome annotation of a related species was used as a reference. The configuration files containing the exact parameters used in the benchmarks can be found at https://github.com/sanger-pathogens/companion-publication.

### Accuracy improvement over stand-alone gene finders

To confirm that *Companion* produces better results than typical standalone gene finders, we annotated the *Plasmodium falciparum* 3D7 (32) chromosome 14 with a set of independent tools, in particular SNAP, AUGUSTUS and RATT using the closely related species *P. reichenowi* CDC (33) as a training set, as well as using *Companion* with a precompiled *P. reichenowi* reference. Comparing completeness and accuracy of the results to the manual *P. falciparum* 3D7 genome annotation, *Companion* outperformed the individual independent tools when run separately (see Supplementary Table S1 for details).

### Whole apicomplexan genome annotation

We also annotated the full version 3 sequence of *P. falciparum* 3D7 using the same *P. reichenowi* reference. The results show that *Companion* consistently annotates genes with high amino acid level sensitivity and specificity (≈98%, Table 1), suggesting that the *Companion* annotation is complete enough to enable a genome wide analysis. We varied several parameters such as use of reference protein or RNA-seq transcriptome evidence as well as the AUGUSTUS score threshold to explore their impact on the *ab initio* gene finding results (Supplementary Table S2). Using more stringent thresholds, the amino acid sensitivity dropped from 98% to about 95%, losing some genes but increasing the specificity to over 99%. As we aim to slightly overpredict to avoid losing gene models, we considered 0.5 to be a good value for the score threshold in practice, given the fact that the specificity is at 98% even with the less stringent parameterization.

**Table 1.** Annotation accuracy evaluation for the example runs on *Leishmania* and *Plasmodium* parasite species

	*L. donovani*	*P. falciparum*	
Extrinsic evidence	Protein	Protein	RNA-seq + protein
Score threshold	0.8	0.5	0.5
# Reference genes	8077	5491	5491
# Predicted genes	8412	5634	5634
Gene level sens	86.60%	92.59%	91.99%
Gene level spec	83.14%	90.24%	89.65%
AA level sens	98.06%	98.07%	98.61%
AA level spec	95.15%	98.34%	98.35%

Please see Supplementary Tables S1 and S2 for complete results for all species.

### Whole kinetoplastid genome annotation

We used the latest version of the *Leishmania donovani* BPK282A1 genome (34) as available from GeneDB as an example to illustrate the annotation of a kinetoplastid parasite (Table 1, column 1). The *Leishmania major* Friedlin version 6 genome (35) was used as a reference. Using these settings, about 86% of the genes were reproduced with perfectly identical coordinates. The remainder of the *L. donovani* genes were predicted with slight coordinate differences, most likely in their upstream gene boundaries (see Supplementary file 1). This is confirmed by substantially higher accuracy at the amino acid level (sensitivity 98%/specificity 95%). 213 loci from the reference were missed in the predicted set (≈2.6%) and 541 predicted loci (6.4%) did not overlap with any gene in the reference. Of the latter, 333 were hypothetical genes; the remainder was annotated with putative functions, for example, encoding surface proteins and ribosomal proteins. It should be noted that some of the non-shared loci are explained by different calls for genes versus pseudogenes in the manual and automatic annotation.

## DISCUSSION

The inherent variation between species and strains of many parasites makes them an obvious target for large-scale sequencing and comparative analysis. Long read sequencing technologies will enable parasitologists to sequence new subspecies or strains to better understand variation in these parasites. Although the assembly process has been improved by recent advances in assembly methods (36,37), the required process to annotate a genome is more difficult to streamline. Tools must be trained individually and do not perform as well separately as they do in combination. Though there are tools to visualize genome annotations (38,39), a user might also appreciate an easier way to assess the quality of the annotation.

To address these needs we have developed the *Companion* web server. It does not only automate the difficult step of *ab initio* gene finding but also improves it. Rather than relying on only one individual tool to correctly call genes in all situations, *Companion* generates a consensus gene set selected from all sources of input evidence (Figure 2). We show that this approach outperforms individual gene finders run separately (see Supplementary Table S1). The annotations generated by *Companion* are rich in information (including GO, products, EC numbers, protein domains, etc.) and ready to submit to public databases to be shared with the community. Annotations can be produced in relatively little time due to the high amount of automation involved. A full annotation run for a *Plasmodium* genome including protein and RNA-seq evidence typically takes about 11 hours, a more streamlined run without these takes about 9 h.

It is important to note that genome annotation in general is not an error-free process. However, the results of the *Leishmania* and *Plasmodium* species benchmarks show that *Companion* delivers largely accurate results. Gene models highly conserved in both the target and the reference are easily transferred, while the sensitivity of the *ab initio* detection of species-specific genes is tunable using score thresholds at a minor expense in specificity. Overall we achieve amino acid level correctness of up to 98–99% (Table 1). We consider this evidence that good results are obtained when suitable, closely related reference species are selected. For more distant references, our experience has shown that the level of success strongly depends on the particular species.

Another notable novel feature in comparison to existing gene finders is the prediction of pseudogenes. Rather than generating shorter or fragmented gene models, *Companion* keeps and marks pseudogenes as potential indicators of biologically relevant changes in the target genome.

The overall aim of *Companion* is to make a first-pass analysis of the annotation as easy as possible. For example, use of the *Companion* server allows a researcher to 1) determine and characterize the level of difference between the target genome and the reference (Venn diagrams and cluster tables, Figure 1F); 2) confirm phylogenetic placement within a set of characterized related species (interactive tree, Figure 1E); 3) generate a summary of gene counts and genome features; 4) assess potential errors in the annotation and 5) inspect high-level synteny of pseudochromosomes (Circos plots, Figure 1C and D). The Circos plots are also helpful to identify rearrangements or misassemblies by providing a chromosome level view of the total genome. In combination, we expect these features to help researchers without a deep bioinformatics background make the most of a given genome sequence using a very low amount of effort.

## CONCLUSION

We have developed a new, integrated software pipeline for the reference-based annotation of parasite genomes and made it available on a public web server. By combining and adapting a multitude of state-of-the-art third-party software, we obtain results that are consistently better than the ones produced by simple runs of stand-alone gene finders. The annotation results can be viewed and queried in the *Companion* web front-end for the essential first-pass analysis. Hand in hand with the improvement of sequencing and assembly technologies, we expect a resource such as *Companion* to enable parasitology researchers to populate databases with often neglected parasite genomes, leading to more consistent and complete analyses in the field.

## Supplementary Material

SUPPLEMENTARY DATA
