# Supplementary material for: Companion: a web server for annotation and analysis of parasite genomes
Source: Nucleic Acids Res. 2016 Apr 21;44(Web Server issue):W29–34. doi: 10.1093/nar/gkw292 (PMC4987884; doi:10.1093/nar/gkw292)
Supplement: SUPPLEMENTARY DATA [file supp_44_W1_W29__index.html]

Companion: a web server for annotation and analysis of parasite genomes — SUPPLEMENTARY DATA 

# *Companion*: a web server for annotation and analysis of parasite genomes

## SUPPLEMENTARY DATA

- SUPPLEMENTARY DATA
- SUPPLEMENTARY DATA
- SUPPLEMENTARY DATA
- SUPPLEMENTARY DATA
