# Supplementary material for: Companion: a web server for annotation and analysis of parasite genomes
Source: Nucleic Acids Res. 2016 Apr 21;44(Web Server issue):W29–34. doi: 10.1093/nar/gkw292 (PMC4987884; doi:10.1093/nar/gkw292)
Supplement: SUPPLEMENTARY DATA [file supp_gkw292_nar-00256-web-b-2016-File008.pdf]

Supplementary Table 1

	RATT	AUGUSTUS	SNAP	*Companion*	*Companion*	*Companion*	*Companion*	*Companion*
annotation sources	RATT	AUGUSTUS	SNAP	RATT+AUGUSTUS	RATT+AUGUSTUS	RATT+AUGUSTUS	AUGUSTUS	RATT + AUGUSTUS
extrinsic evidence				protein	protein	protein	RNA-seq	RNA-seq + protein
score threshold				0.5	0.7	0.8	0.5	0.5
Reference genes	786	786	786	786	786	786	786	786
Prediction genes	768	585	927	780	770	762	761	783
**Gene level**								
sensitivity partial	748/786 95.17%	575/786 73.16%	702/786 89.31%	765/786 97.33%	758/786 96.44%	751/786 95.55%	746/786 94.91%	768/786 **97.71%**
specificity partial	757/768 98.57%	573/585 97.95%	761/927 82.09%	761/780 97.56%	754/770 97.92%	747/762 **98.03%**	743/761 97.63%	765/783 97.70%
sensitivity complete	723/786 91.98%	541/786 68.83%	284/786 36.13%	740/786 94.15%	734/786 93.38%	727/786 92.49%	661/786 84.10%	740/786 **94.15%**
specificity complete	730/768 95.05%	541/585 92.48%	284/927 30.64%	740/780 94.87%	734/770 95.32%	727/762 **95.41%**	661/761 86.86%	740/783 94.51%
**MRNA level**								
sensitivity partial	757/775 97.68%	580/775 74.84%	704/775 90.84%	766/775 98.84%	759/775 97.94%	752/775 97.03%	744/775 96.00%	769/775 **99.23%**
specificity partial	757/768 98.57%	573/585 97.95%	755/927 81.45%	753/773 97.41%	746/763 97.77%	739/755 **97.88%**	735/754 97.48%	757/776 97.55%
sensitivity complete	735/775 94.84%	545/775 70.32%	285/775 36.77%	743/775 95.87%	737/775 95.10%	730/775 94.19%	663/775 85.55%	743/775 **95.87%**
specificity complete	735/768 95.70%	542/585 92.65%	285/927 30.74%	736/773 95.21%	730/763 95.67%	723/755 **95.76%**	657/754 87.14%	736/776 94.85%
**CDS level**								
sensitivity partial	2096/2170 96.59%	1290/2170 59.45%	1911/2170 88.06%	2114/2170 97.42%	2086/2170 96.13%	2069/2170 95.35%	1964/2170 90.51%	2121/2170 **97.74%**
specificity partial	2126/2164 98.24%	1256/1281 98.05%	2066/3775 54.73%	2016/2054 98.15%	1988/2013 98.76%	1971/1994 **98.85%**	1916/1972 97.16%	2024/2060 98.25%
sensitivity complete	2060/2170 94.93%	1244/2170 57.33%	1245/2170 57.37%	2074/2170 95.58%	2048/2170 94.38%	2032/2170 93.64%	1862/2170 85.81%	2079/2170 **95.81%**
specificity complete	2060/2164 95.19%	1215/1281 94.85%	1183/3775 31.34%	1986/2054 96.69%	1960/2013 97.37%	1944/1994 **97.49%**	1814/1972 91.99%	1991/2060 96.65%
reference	2060/2111 97.60%	1215/1264 96.10%	1168/2028 57.60%	1986/2022 98.20%	1960/1993 **98.30%**	1944/1977 **98.30%**	1814/1925 94.20%	1991/2031 98.00%
prediction	2060/2128 96.80%	1215/1265 96.00%	1168/3220 36.30%	1986/2017 98.50%	1960/1988 **98.60%**	1944/1971 **98.60%**	1814/1938 93.60%	1991/2026 98.30%
**Nucleotide level**								
sensitivity	98.28%	68.40%	91.66%	96.89%	95.79%	94.21%	92.15%	**96.98%**
specificity	98.62%	98.68%	94.50%	98.73%	**98.87%**	98.87%	98.64%	98.77%
**Amino Acid level**								
sensitivity	98.00%	68.39%	91.44%	96.87%	95.77%	94.19%	92.13%	**96.96%**
specificity	98.35%	98.68%	94.27%	98.72%	**98.86%**	98.85%	98.62%	98.75%
Annotation edit distance	0.002	0.001	0.046	0	0	0.001	0.002	0.001
F1 score	0.998	0.999	0.953	1	1	0.999	0.998	0.999
Matching coefficient	0.997	0.998	0.936	0.999	0.999	0.999	0.997	0.999
Loci total	771	771	799	780	777	776	778	779
Loci shared	741	570	711	752	745	738	731	755
Loci reference only	19	189	6	8	15	22	28	**5**
Loci prediction only	11	12	82	20	17	**16**	19	19

RNA-seq reads were mapped to the genomic sequence using TopHat v2.0.8b and assembling transcripts using Cufflinks v2.2.1
