# Supplementary material for: Companion: a web server for annotation and analysis of parasite genomes
Source: Nucleic Acids Res. 2016 Apr 21;44(Web Server issue):W29–34. doi: 10.1093/nar/gkw292 (PMC4987884; doi:10.1093/nar/gkw292)
Supplement: SUPPLEMENTARY DATA [file supp_gkw292_nar-00256-web-b-2016-File009.pdf]

Supplementary Table 2

	L. donovani		*P. falciparum*									
annotation sources	RATT + AUGUSTUS		RATT + AUGUSTUS		RATT + AUGUSTUS		RATT + AUGUSTUS		AUGUSTUS		RATT + AUGUSTUS	
extrinsic evidence	protein		protein		protein		protein		RNA-seq		RNA-seq + protein	
score threshold	0.8		0.5		0.7		0.8		0.5		0.5	
Reference genes	8077		5491		5491		5491		5491		5491	
Prediction genes	8412		5634		5415		5311		5496		5634	
**Gene level accuracy**												
sensitivity partial	7826/8077	96.89%	5329/5491	97.05%	5239/5491	95.41%	5182/5491	94.37%	5212/5491	94.92%	5333/5491	**97.12%**
specificity partial	7830/8412	93.08%	5301/5634	94.09%	5209/5415	96.20%	5157/5311	**97.10%**	5173/5496	94.12%	5307/5634	94.20%
sensitivity complete	6995/8077	86.60%	5084/5491	**92.59%**	5033/5491	91.66%	5003/5491	91.11%	4555/5491	82.95%	5051/5491	91.99%
specificity complete	6994/8412	83.14%	5084/5634	90.24%	5033/5415	92.95%	5003/5311	**94.20%**	4555/5496	82.88%	5051/5634	89.65%
**mRNA level accuracy**												
sensitivity partial	7751/7965	97.31%	5314/5364	99.07%	5224/5364	97.39%	5173/5364	96.44%	5190/5364	96.76%	5316/5364	**99.11%**
specificity partial	7754/8295	93.48%	5221/5568	93.77%	5137/5349	96.04%	5087/5245	**96.99%**	5093/5430	93.79%	5227/5568	93.88%
sensitivity complete	6967/7965	87.47%	5098/5364	**95.04%**	5045/5364	94.05%	5015/5364	93.49%	4569/5364	85.18%	5063/5364	94.39%
specificity complete	6966/8295	83.98%	5048/5568	90.66%	4997/5349	93.42%	4967/5245	**94.70%**	4519/5430	83.22%	5014/5568	90.05%
**CDS level accuracy**												
sensitivity partial	7760/8098	95.83%	13919/14148	98.38%	13651/14148	96.49%	13496/14148	95.39%	13387/14148	94.62%	13939/14148	**98.52%**
specificity partial	7778/8632	90.11%	13453/14174	94.91%	13200/13584	97.17%	13043/13315	**97.96%**	13005/13912	93.48%	13483/14235	94.72%
sensitivity complete	7015/8098	86.63%	13590/14148	**96.06%**	13375/14148	94.54%	13243/14148	93.60%	12619/14148	89.19%	13575/14148	95.95%
specificity complete	7014/8632	81.26%	13198/14174	93.11%	12992/13584	95.64%	12860/13315	**96.58%**	12270/13912	88.20%	13184/14235	92.62%
coding sensitivity	7014/7769	90.30%	13198/13517	97.60%	12992/13256	98.00%	12860/13093	**98.20%**	12270/13040	94.10%	13184/13529	97.40%
coding specificity	7014/7993	87.80%	13198/13505	97.70%	12992/13228	98.20%	12860/13060	**98.50%**	12270/13261	92.50%	13184/13578	97.10%
**Nucleotide level accuracy**												
sensitivity		98.15%		98.08%		96.35%		95.36%		94.70%		**98.63%**
specificity		95.24%		98.35%		98.93%		**99.15%**		98.25%		98.36%
**Amino Acid level accuracy**												
sensitivity		98.06%		98.07%		96.34%		95.35%		94.68%		**98.61%**
specificity		95.15%		98.34%		98.92%		**99.14%**		98.24%		98.35%
Annotation edit distance		0.015		0.001		0.001		0.001		0.002		0.001
F1 score		0.985		0.999		0.999		0.999		0.998		0.999
Matching coefficient		0.971		0.998		0.998		0.998		0.996		0.998
Loci total		8501		5611		5479		5426		5582		5604
Loci shared		7747		5216		5133		5084		5081		5221
Loci reference only		213		48		134		184		166		**43**
Loci prediction only		541		347		212		**158**		335		340

RNA-seq reads were mapped to the genomic sequence using TopHat v2.0.8b and assembling transcripts using Cufflinks v2.2.1
