# Supplementary material for: Companion: a web server for annotation and analysis of parasite genomes
Source: Nucleic Acids Res. 2016 Apr 21;44(Web Server issue):W29–34. doi: 10.1093/nar/gkw292 (PMC4987884; doi:10.1093/nar/gkw292)
Supplement: SUPPLEMENTARY DATA [file supp_gkw292_Supplementary_File_1.pdf]

Companion: a web server for annotation and analysis of parasite genomes

Sascha Steinbiss, Fatima Silva-Franco, Brian Brunk, Bernardo Foth, Christiane Hertz-Fowler, Matthew Berriman and Thomas Dan Otto

Supplementary File 1

1. PSEUDOGENE DETECTION

The pseudogene assignment approach is similar to the one used by PseudoPipe (1), but uses more modern and efficient tools to achieve its goal. Reference protein sequences are aligned to the target DNA sequence, allowing for frame shifts. This is done using LAST (2) after preprocessing the input sequences with tantan (3) to reduce the amount of unspecific matching due to repeats.

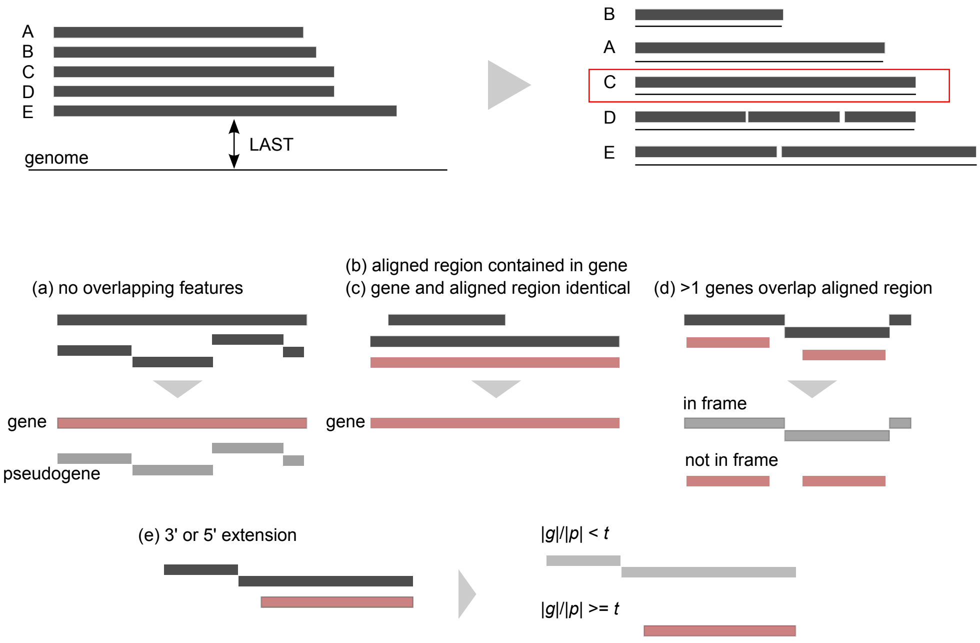

Of all proteins aligning to the same target locus, the median length one is selected to avoid excessively long or short homologous matches from species with potentially different levels of curation (match C in the example above).

The selected match is then reconciled with existing gene models: (a) no gene is predicted at the locus, keep match and label as gene or pseudogene, matches (b) contained in or (c) identical to existing genes are not considered potential pseudogenes, (d) short genes overlapping the frame-shifted match are subsumed by a pseudogene if they agree with the frames of the pseudogenic exons they overlap, and (e) genes for which a protein match suggests a possible 5’ or 3’ extension are converted to pseudogenes if the extended region exceeds a given proportion of the gene length.

2. GENE MODEL POST-PROCESSING

Some parasite species show uncommon characteristics requiring special attention during gene finding. For example, trypanosomatids such as *Trypanosoma* and *Leishmania* show polycistronic transcription (4), a process involving groups of genes on the same strand

(polycistronic transcription units or PTUs), each separated by strand-switch regions (SSR). To eliminate spurious false positive gene predictions on the strand opposite of the PTU in question, we have added an optional post-processing step ('PTU smoothing'). It can be described as follows: Let $strand(g)$ of a gene g be 1 if g is located on the forward strand, and -1 if g is located on the reverse strand. Each gene g_j of n total genes in a pseudochromosome can then be assigned a *neighborhood score* ss with *neighborhood radius* v (default $v = 6$) as follows:

$$ss(g_i, v) = \sum_{j=\max(1,i-v)}^{\min(i+v,n)} strand(g_j)$$

Hence every gene in a forward strand neighborhood is assigned a positive score, and a gene in a reverse strand neighborhood is assigned a negative score, with the score of genes in SSRs approaching zero from either side. All genes showing disagreement between their strand annotation and their neighborhood score, i.e. genes for which $\operatorname{sgn}\big(ss(g_i, v)\big) \neq strand(g_i)$, are removed.

3. SANITIZATION AND VALIDATION

To minimize the impact of low quality or erroneous gene models on the submission process, we have implemented multiple sanitizing steps at various points in the pipeline, making sure that, for example, features annotated as genes have intact protein translations, do not intersect with gaps or have consistent coordinates across the transcript and CDS features. Problematic features will be automatically cleaned up as required. Finally, an HTML report is generated, listing all potentially remaining problematic features as well as the nature of their requirement violations. Such a report is useful for a curator to quickly assess the potentially required amount of manual editing and to act as a checklist for the curation process.

4. STAND-ALONE VERSION

The stand-alone version can be run efficiently on powerful desktop machines as well as large compute clusters (LSF, SGE, SLURM, …). As *Companion* depends on a multitude of third-party software, we also provide a Docker container[1] encapsulating all external dependencies, including software binaries and most of the freely redistributable data files[2]. If the Docker container is used, the only additional software requirements for Linux users are the Nextflow workflow engine[3] and the Java 7 JRE, both of which are easy to install on modern distributions. Users of non-Linux platforms such as Mac OS X also require the boot2docker virtual machine[4]. The use of a Docker container comes with only a minimal speed tradeoff compared to a customized installation (5), and its use as a reference execution environment also ensures that results stay reproducible (6, 7) as long as the same container is used across invocations.

It should be noted that such stand-alone use requires preparation and preprocessing of a reference data set, which may include additional organisms besides the one used as the annotation reference. These organisms can be organized into subgroups, e.g. for a kinetoplastid reference, the *Trypanosoma* and *Leishmania* genera can be separated while still being able to make use of orthology information across all species. Reference annotations and sequences can be imported from appropriately formatted FASTA, GAF and GFF3 files. For the species available as references on the web server, the pre-processed reference data

---

[1] http://www.docker.com

[2] https://hub.docker.com/r/satta/companion/ (~2.5GB)

[3] http://www.nextflow.io

[4] http://boot2docker.io

sets are also available for download[5] to use with the stand-alone tool. We also provide a brief on-line documentation of the reference preparation process on the *Companion* GitHub wiki[6].

5. EVALUATION MEASURES FOR ANNOTATION COMPARISON

To assess agreement between prediction and reference annotations, we employ the notions of 'partial' matches, for which two features have to overlap to produce a match, and 'complete' matches, requiring exactly identical coordinates on the annotated sequence. The number of partial and complete matches is used to compute sensitivity and specificity values on the gene, transcript and CDS/exon levels. We also calculated nucleotide and amino acid level specificity and sensitivity to provide a more fine-grained assessment of accuracy.

A custom implementation was used to calculate these values based on GFF3 files, considering both non-coding and coding genes. As a second source of results, we used the existing ParsEval software (8) which gave similar results but only considers protein-coding genes. On the other hand it also calculates other common measures like matching coefficient, annotation edit distance and F1 score (9, 10). They are also given in supplementary table 1 and 2 for all comparison runs.

5. GENE COORDINATE DEVIATIONS

To determine the nature and extent of the imperfect gene model predictions in the Leishmania donovani use case, we plotted the deviation of the predicted start and end position of the gene compared to the respective position in the manually annotated reference. Most of the differences are found in the start positions (n=565), much less in the end positions (n=140). There was no convincing evidence for a preference towards elongation or shortening of gene models upstream. Only in infrequent extreme cases did the difference extend beyond 1Kb in either direction.

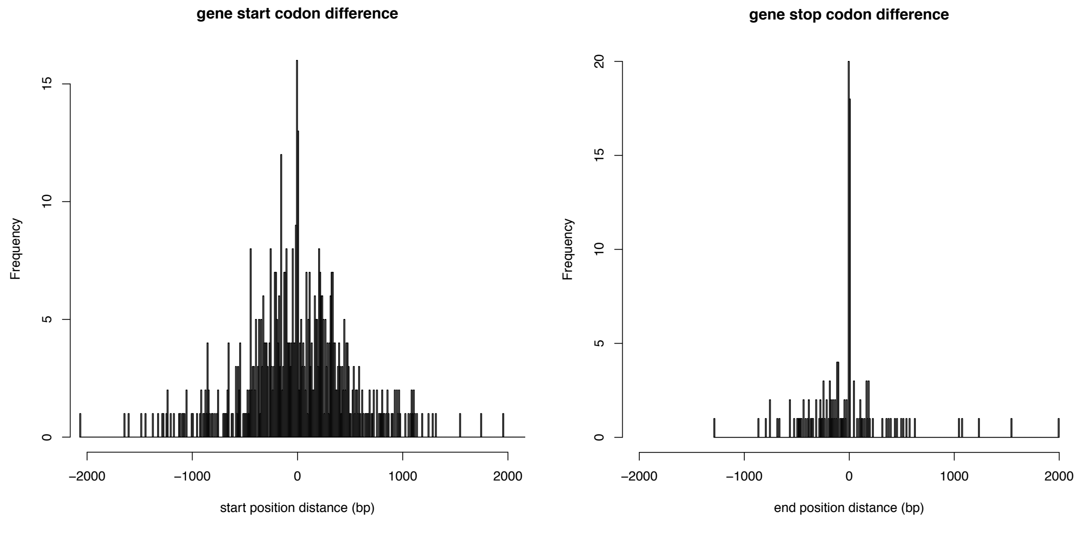

6. SOFTWARE FOUNDATIONS

To perform efficient and robust processing of genomic sequences and annotations, *Companion* is built on its lowest level on the software infrastructure provided by the *GenomeTools* toolkit (11). This includes the stand-alone executable `gt` as well as custom

[5] ftp://ftp.sanger.ac.uk/pub/project/pathogens/companion

[6] https://github.com/sanger-pathogens/companion/wiki/Preparing-reference-data-sets

scripts written in the Lua programming language (12) using the GenomeTools application programming interface (API).

BIBLIOGRAPHY

1. Zhang,Z., Carriero,N., Zheng,D., Karro,J., Harrison,P.M. and Gerstein,M. (2006) PseudoPipe: an automated pseudogene identification pipeline. *Bioinformatics*, **22**, 1437–9.

2. Kiełbasa,S.M., Wan,R., Sato,K., Horton,P. and Frith,M.C. (2011) Adaptive seeds tame genomic sequence comparison. *Genome Res.*, **21**, 487–93.

3. Frith,M.C. (2011) A new repeat-masking method enables specific detection of homologous sequences. *Nucleic Acids Res.*, **39**, e23.

4. Campbell,D.A., Thomas,S. and Sturm,N.R. (2003) Transcription in kinetoplastid protozoa: why be normal? *Microbes Infect.*, **5**, 1231–1240.

5. Di Tommaso,P., Palumbo,E., Chatzou,M., Prieto,P., Heuer,M.L. and Notredame,C. (2015) The impact of Docker containers on the performance of genomic pipelines. *PeerJ*, **3**, e1273.

6. Piccolo,S.R., Lee,A.B. and Frampton,M.B. (2015) Tools and techniques for computational reproducibility. *bioRxiv*, 10.1101/022707.

7. Boettiger,C. (2015) An introduction to Docker for reproducible research. *ACM SIGOPS Oper. Syst. Rev.*, **49**, 71–79.

8. Standage,D.S. and Brendel,V.P. (2012) ParsEval: parallel comparison and analysis of gene structure annotations. *BMC Bioinformatics*, **13**, 187.

9. Blair,D.C. (1979) Information Retrieval, 2nd ed. C.J. Van Rijsbergen. London: Butterworths; 1979: 208 pp. Price: $32.50. *J. Am. Soc. Inf. Sci.*, **30**, 374–375.

10. Burset,M. and Guigó,R. (1996) Evaluation of gene structure prediction programs. *Genomics*, **34**, 353–67.

11. Gremme,G., Steinbiss,S. and Kurtz,S. (2013) GenomeTools: a comprehensive software library for efficient processing of structured genome annotations. *IEEE/ACM Trans. Comput. Biol. Bioinforma.*, **10**, 645–56.

12. Ierusalimschy,R., de Figueiredo,L.H. and Filho,W.C. (1996) Lua—An Extensible Extension Language. *Softw. Pract. Exp.*, **26**, 635–652.
